# Comparative study between school performance on first grade children and suppression of otoacoustic transient emission

**DOI:** 10.1016/S1808-8694(15)30760-6

**Published:** 2015-10-19

**Authors:** Miguel Luiz de Sant'Ana Angeli, Clemente Isnard Ribeiro de Almeida, Patrícia M. Sens

**Affiliations:** 1 M. Sc Student - Graduate Program in Otorhinolaryngology - Faculdade de Ciências Médicas da Santa Casa de Misericórdia - São Paulo, MD. ENT; 2 Full Emmeritus Professor - Medical School of Jundiaí, Graduate Program Professor - Faculdade de Ciências Médicas da Santa Casa de Misericórdia de São Paulo, MD. ENT; 3 Graduate Student in Otorhinolaryngology - Faculdade de Ciências Médicas da Santa Casa de Misericórdia de São Paulo, MD. ENT

**Keywords:** learning disabilities, otoacoustic emissions, laterality, medial olivocochlear system, contralateral supression

## Abstract

School learning can be hampered if there are defects on the central auditory process. Since those with auditory deficiency can be rehabilitated, it is fundamental that we identify them. Otoacoustic emissions test has low cost and operational ease. Study design: clinical and experimental.

**Aim:**

to study the relationship between school learning and transient otoacoustic emission suppression by contralateral stimuli.

**Material and Methods:**

39 individuals, from 7 to 12 years of age were evaluated, 19 (48.7%) with good school performance and 20 (51.3%) poor performers.

**Results:**

A transient otoacoustic emission suppression failure for contralateral acoustic stimuli was more frequently found among children with poor school performance. We established a value of 1.6 dB SPL for emission reduction that characterized those children as belonging to the poor learning performance group: sensitivity 65%, specificity 72,2%, accuracy of 68.4%, positive predictive value of 72.2%.

**Conclusion:**

The contralateral emission suppression test of the right ear can be predictive of school difficulties in individuals from six to twelve years of age.

## INTRODUCTION

The auditory function and most specifically that of auditory communication have been extensively studied. The hypothesis that losses in auditory perception may be associated with the difficulty in learning the sound-symbol relationships which make up the very basis of phonetic rules, and that there is a relationship between acquiring reading and writing skills and the underlying speech and hearing skills have been growing in number of participants. Some researchers have studied the relationships between problems associated with temporal processing of auditory stimuli and losses of some speech and hearing skills and phonemic segmentation. First grade schools are true laboratories for cognition evaluation. Apparently, a number of factors interfere on the school performance of children; among them we have the functional integrity of the auditory system. Identifying this deficiency can be relevant in the process of rehabilitating these children.

Hearing is highly complex, when seen from its peripheral component, however well understood in most of its aspects. Notwithstanding, the central physiology associated with auditory communication still is an open field, both for basic and for applied research. As to the efferent pathways, Kimura^1^ noticed that when auditory stimuli are presented in a dichotomist fashion, the ipsilateral pathways are suppressed by their contralateral counterparts. According to her, verbal auditory information that reaches the right ear would go to the left cerebral hemisphere, which is dominant for verbal language, by means of the contralateral auditory pathways, going through the commissure of the corpus callosum.

In 1978, Kemp^2^, concluded that the sound generated by the physiological activities of the outer hair cells is then taken through the middle ear to the external acoustic meatus, where its emission can be recorded. Since then, many papers have discussed the suppression of otoacoustic emissions in human beings by means of a contralateral stimulation.^3^, ^4^, ^5^, ^6^, ^7^

This phenomenon is due to a stimulation of efferent synapses of outer hair cells^7^, which would occur through the olivo-cochlear bundle and would depend on descending pathways originating on cortical and sub-cortical regions. Thus, emission suppression could be influenced by the most varied central pathological situations.

The possibility of assessing otoacoustic emissions has helped the semiology of hearing peripheral organs because it is an objective, sensitive and specific method. Observing a reduction in the otoacoustic emission amplitudes evoked by the contralateral sound stimulus, it was considered that this phenomenon may be used to assess not only the acoustic nerve, but also the central efferent pathways of the auditory system.

Anatomical and physiological evidence state that the function of both ears is interdependent and coordinated by the efferent neural pathways, which connects one side of the auditory system to the other side, through the medial and lateral components of the olivocochlear system. The medial olivocochlear bundle is made up of approximately 80% of crossed nerve fibers and 20% of ipsilateral never fibers, it projects its nerve endings mainly to the contralateral cochlea, ending just below the outer hair cells.

The lateral olivocochlear bundle, which is made up of about 90% of ipsilateral nerve fibers and 10% of crossed fibers, projects its endings mainly to the ipsilateral inner hair cells, ending at the efferent radial auditory endings that leave these cells.

Many researchers have shown that the contralateral inhibition of emissions is a neural phenomenon, caused by the efferent system.^8^, ^9^, ^10^ In their studies, they measured transient and distortion product otoacoustic emissions, with and without a narrow band contralateral stimulus to activate the efferent olivocochlear nervous system, and the results led their authors to consider the test a useful tool in the set of procedures for the diagnosis of retrocochlear disorders.^8^, ^9^, ^10^

The current paper aims at checking the lack of otoacoustic emission inhibition on the right ear by a contralateral stimulus, which can be used as screening tool or when the physician suspects of auditory processing dysfunction seen when children between six and twelve years of age underperform at school.

## MATERIALS AND METHODS

This study was submitted and approved by the Ethics in Research Committee of our institution and approved under protocol no. 390/04. A municipal first grade school of a neighboring town to São Paulo was chosen for the study and the guardians of the participating children signed and informed consent form. All regularly enrolled students who fit the methodology criteria were included. The children included in the study were divided between the ones with the best and those with the worst school performance in their classes.

We assessed 39 children with ages between 6 and 12 years, 16 (41.1%) were females and 23 (58.9%) were males. Among them, 19 (48.7%) had good school performance and 20 (51.3%) had poor performance.

The following inclusion criteria were observed: no family history of hereditary hearing deficiency, no family history of repetition otitis, no use of ototoxic medication, no exposure to occupational noise of hearing thresholds up to 25 dBHL in the frequencies of 250, 500, 1000, 2000, 3000, 4000, 6000, and 8000 Hz, bilaterally, type A immitance curves and bilateral presence of contralateral stapedial acoustic reflex, with normal otoscopic examination.

Exclusion criteria were: psychological problems, uncorrected visual deficiency, hearing deficit, neurological dysfunction or low IQ.

### Evaluation procedures

In the clinical history, we questioned them on the prior existence of otologic diseases, the use of ototoxic medication, tinnitus, other diseases and complaints and current ear problems. Physical exam involved facial inspection, ears, external auditory meatus and tympanic membranes. Audiologic exams were: tonal audiometry, speech understanding, speech recognition threshold, immitanciometry and tympanometry, stapes muscle threshold reflex at 500, 1000, 2000, and 4000 Hz and reflex fatigue study at 500 and 1000 Hz. In order to collect otoacoustic emissions, we used the Echoport ILO 288 system, from the English Otodynamics, sold in Brazil by Siemens. We used linear and non-linear clicks.

In order to capture transient otoacoustic emissions, we used non-linear sound clicks, three of them in one polarity and one of inverse polarity, with amplitude three times higher than that of the first, lasting for 100ms, with intensities between 70 and 80 dBSPL, in a total number of 3000 stimuli accepted and also linear clicks.

Whether or not the child had suppression was checked with linear and non-linear clicks, depending on the type of the original stimulus. The frequencies encompassed by the stimulus were between 500 Hz and 4000 Hz. The clicks presented as stimuli were condensed, in such a way that the first part of the stimulus pushes the tympanic membrane medially and, consequently, the base membrane is moved outwardly.

For the contralateral stimuli, we used a concurrent narrow band noise on the contralateral ear. The noise intensity should be of approximately 10 dB above the sound stimuli that caused otoacoustic emissions, however below the stapes reflex level on the tested ear.

For contralateral stimulation we used an AC 33 audiometer, from Interacoustics, a Danish device, sold in Brazil by Siemens.

To try and achieve transient otoacoustic emissions suppression, the noise range was fixed between 750 Hz and 3000Hz. The suppressive noise was presented at approximately 9ms after the onset of the stimulus used to acquire the otoacoustic emissions.

### Statistical Analysis

All variables were analyzed descriptively (table 1). For the quantitative variables this analysis was carried out through observing the minimum and maximum values, calculating the averages, standard deviations and median values. For the qualitative variables we calculated the absolute and relative frequencies.

**Table 1:** Otoacoustic emissions with and without suppression by contralateral stimuli in 39 children.

Case	Gender	Age	Performance	Right ear without suppression	Right ear with suppression	Left ear without suppression	Left ear with suppression	With and without suppression difference for the right ear	With and without suppression difference for the left ear
1	M	7	Good	13	12.3	18.5	17	0.7	1.5
2	M	10	Poor	15.4	14.3	18.8	16.8	1.1	2
3	M	9	Poor	17.5	16.8	19.4	18.9	0.7	0.5
4	F	10	Poor	26.2	24.3	25.5	22.2	1.9	3.3
5	F	9	Poor	25.3	23.4	26.6	24.8	1.9	1.8
6	F	7	Poor	17.5	17.5	18.5	18.1	0	0.4
7	M	7	Poor	24.9	24.7	20.4	18.2	0.2	2.2
8	M	7	Poor	15.5	13.5	17.9	17.1	2	0.8
9	M	7	Poor	15.8	15.4	18.4	17.3	0.4	1.1
10	F	6	Poor	23.7	22.4	28.4	25.8	1.3	2.6
11	M	9	Poor	23	21.2	24	22.6	1.8	1.4
12	M	7	Poor	16.9	15.8	18.1	15.3	1.1	2.8
13	F	9	Poor	19.1	16.9	17.1	13.7	2.2	3.4
14	M	10	Poor	20.9	20.7	23.6	19.7	0.2	3.9
15	M	9	Poor	20.2	19.2	20.6	18.7	1	1.9
16	M	9	Poor	11.5	10.8	10.8	8.3	0.7	2.5
17	M	7	Poor	24.9	23.9	23.4	21.6	1	1.8
18	F	6	Poor	28.6	26.3	28.8	25.7	2.3	3.1
19	F	10	Poor	24.5	22.6	20.1	17.9	1.9	2.2
20	M	12	Poor	24.7	24.4	25.8	23.3	0.3	2.5
21	M	10	Poor	20.7	12.5	19.5	16.5	8.2	3
22	M	8	Poor	28.6	27.4	21.8	20.3	1.2	1.5
23	M	8	Good	14.3	12.4	15.6	13.4	1.9	2.2
24	F	7	Good	24	22	18.6	14.3	2	4.3
25	F	9	Good	13.6	12.3	17.1	13.6	1.3	3.5
26	M	8	Good	19.8	18.1	22.2	20.4	1.7	1.8
27	F	8	Good	15	12.6	18	14.2	2.4	3.8
28	F	10	Good	14.6	12.7	16	14.5	1.9	1.5
29	F	9	Good	16.8	15	20.5	17.7	1.8	2.8
30	F	10	Good	19.9	16.6	23.7	20.4	3.3	3.3
31	F	10	Good	21.8	19.4	22.1	19.3	2.4	2.8
32	F	9	Good	15.4	13.6	20.5	17	1.8	3.5
33	F	10	Good	18.1	18	19.3	14.6	0.1	4.7
34	M	11	Good	22.6	20.4	19.4	18.5	2.2	0.9
35	M	9	Good	29.3	27.5	29.4	27.7	1.8	1.7
36	M	10	Good	16.8	16.3	13	11	0.5	2
37	M	9	Good	11.1	10	10.6	8.9	1.1	1.7
38	M	10	Good	19.4	17.4	23.2	21.4	2	1.8
39	M	9	Good	18.4	16.2	18.9	18.4	2.2	0.5

In order to analyze the equality-between-the-groups hypothesis, we used the t Student test, and as for the assumption of data normality we used the Mann-Whitney non-parametric test for independent samples.

In order to test for group homogeneity in relation to the proportions we used the chi-squared test. In order to assess if some measure carried out could predict poor school performance we used the logistics regression model. We obtained a cutting point for the measure and calculated the efficiency indices. We used 5% as significance level for the tests.

## RESULTS

Our results are shown on Table 1, Table 2, Table 3.

**Table 2:** Emission values in decibels (dB), mean, standard deviation, median, minimum and maximum of the differences between the measures with and without suppression on the ears assessed according to performance.

Ear	Performance	n	Mean	sd	Median	Minimum	Maximum	p*
	Good	18	1.73	0.76	1.85	0.10	3.30	
Right	Poor	21	1.16	0.73	1.10	0.00	2.30	0.039
	Good	18	2.46	1.18	2.10	0.50	4.70	
Left	Poor	21	2.09	0.96	2.10	0.40	3.90	0.429

* Likelihood descriptive level for the Mann-Whitney non-parametric test

**Table 3:** Poor performance likelihood values estimated with the logistics regression model.

Measure	Likelihood
0	0.836
1	0.641
2	0.384
3	0.179

We have noticed that the performance groups were not different as far as age and gender were concerned. They did not bear significant differences in the left ear measures with and without suppression (Table 2).

On the right ear, there were statistically significant differences between the different measures with and without suppression; the poor performance group had significantly lower values when compared to the group with good performance (Table 2).

**Graph 1:**
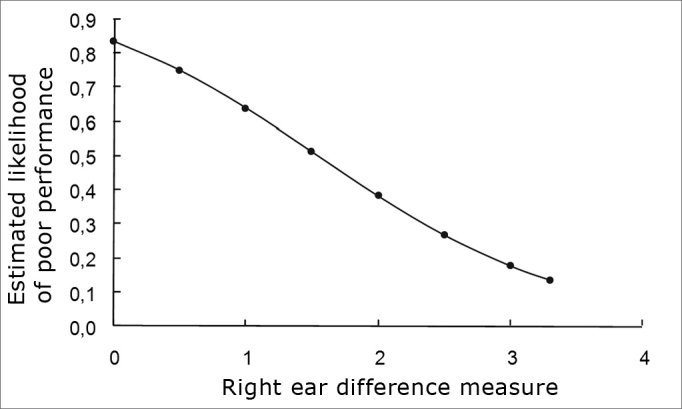
Logistics regression model. Suppression failure in decibels.

Analyzing the different variables with and without suppression on the right ear by means of a logistic regression11, we noticed that such variable was associated with school performance (p=0.034). Graph 1 shows the likelihood of poor performance associated with the values of differences measured with and without suppression on the right ear.

On Table 3 we see some poor performance likelihood values estimated by means of the logistics regression model for some values of different measures with and without suppression for the right ear.

Thus, a child with a measure value equal to zero has an 83% likelihood of having poor performance, for a measure value equal to 3, this likelihood falls down to 18%.

Through logistics regression model we can find a cutting point from which we have a greater chance of poor performance.

Such value is equal to 1.6 and gives us a sensitivity of 65.0%, specificity of 72.2%, accuracy of 68.4%, positive predictive value of 72.2% and negative predictive value of 65%.

The children with differences below 1.6 have 4.83 (confidence interval at 95%: 1.21; 19.22) fold higher chance of poor performance when compared to those who presented differences above 1.6.

Future studies with larger samples are necessary in order to validate this method as being useful for the screening to identify those with auditory processing dysfunction among children with learning disorders.

## DISCUSSION

Poor performance at school is a source of great concern to parents and teachers. The causes for this deficiency are numerous, such as social, nutritional, family, teaching system, even problems intrinsic to children such as neurologic, psychiatric, psychological, visual and hearing problems, besides a lack of maturation or dysfunction of the cognitive nervous system.

Children with learning disabilities end up having a lower intellectual and social development than their conditions allow. To locate the cause of this deficiency and to overcome it can change their lives. When we observe a reduction in the otoacoustic emission amplitude values evoked by a contralateral sound stimulus, the possibility was considered that such phenomenon cold be used to assess, in a practical way, not only the acoustic nerve, but also the auditory system efferent central pathways, certainly connected with auditory communication.

Elementary schools are true laboratories where cognition is assessed. The present investigation aims at comparing the otoacoustic emission amplitude values evoked by the contralateral sound stimulus of the students ranked in first and last in performance from an elementary school at the State of São Paulo.

The efferent pathways were identified and studied by numerous authors^3^^,^^4^^,^^5^^,^^6^^,^^12^^,^^13^, and contralateral inhibitions suppression was initially studied by Collet^8^ and later confirmed by many others^10^^,^^14^^,^^15^. In 1999, Pialarassi^10^ studied the suppression of transient and distortion product otoacoustic emissions with contralateral stimulus by a narrow band noise in 48 individuals with normal hearing and 9 individuals with retrocochlear disease. In the normal group there was significant suppression of otoacoustic emissions. In the group with the disease, sometimes they found mild suppression and sometimes it did not occur, and sometimes there was intensification. The results show that the otoacoustic emission suppression with contralateral stimuli is a useful tool in the set of procedures used to diagnose retrocochlear auditory disorders.

Laterality is an important factor for the satisfactory performance of multiple body functions, including hearing and auditory processing. Research^16^ have shown that the left brain hemisphere prevails over the right side in speech auditory processing; while the right side prevails in the processing of tones and musical stimuli. Kimura1 states it in a basic research paper published in 1963, that the verbal auditory information presented to the right ear come to the left hemisphere, which is dominant for verbal language, through the contralateral auditory pathways, going through the commissure of the corpus callosum. In the sample analyzed, we could learn that the auditory inhibition disorder manifestation by a simultaneous contralateral stimulus manifested clearly and significantly when hearing was assessed on the right ear.

The meaning of this observation is, to start with, an indication that if this test is used in the study of auditory processing disorders, it must be made with a stimulus being presented to the right ear and a competitive sound in the left contralateral ear. The same thinking must be used when we rehabilitate individuals with auditory processing disorders, especially those that have concurrent auditory impairment, giving preference to amplification and rehabilitation stimuli in the right ear.

Tests such as SSW were applied to identify auditory processing problems in school-age children. In 1984 Berrick et al17 studying the performance of 93 children without learning complaints and 97 children with learning disabilities, in the age range between 8 and 11 years by the SSW test, observed that the children without school complaints presented a statistically significant better performance when compared to those children with learning disorders, stressing the usefulness of the SSW test in the hearing function of the children with learning difficulties. The same was observed by Almeida^18^ using the PSI test adapted to Portuguese by Almeida^19^. These tests proved efficient and in certain ways objective; however, their application requires complex equipment. Both SSW and PSI are screening tests which are not specific for the type of auditory processing deficiency; however, very safe in relation to the results. Later studies must be applied to a similar group, with SSI and SSW tests, besides the suppression failure study in order to validate the importance of this research in the diagnosis of processing dysfunction, as it was stressed in the introduction, hearing processing is not the only cause of learning disorders.

Musiek and other authors^20^^,^^21^^,^^22^ observed that central auditory processing disorders are, usually, cortical or sub-cortical dysfunctions that can be secondary to maturation delays or morphological abnormalities.

The possibility of using a simple screening test for children with low school performance in an attempt to identify those with processing problems, is important to indicate the need to refer these students to more complex tests and finally guide their rehabilitation.

Our study showed very stimulating results as to the chances of obtaining a low and efficient test with a reasonable predictive value to identify auditory processing potential disorders. We need longitudinal tests with larger cohorts and broader samples to assess test specificity and sensitivity. The confirmation of learning disorders with children that have previously been considered of risk may turn this test into an accurate and mandatory instrument in the assessment of pre-school age children.

Knowing that the children with auditory processing dysfunction, when properly diagnosed, may be rehabilitated without speech and hearing training, changing not only their immediate school performance, but also the life style and quality of these children in the long run is a powerful stimulus to carry out new studies in this filed.

Future studies with larger series and comparing the results with SSI and SSW are necessary to validate this method as useful in the screening of children with auditory processing disorders among those with learning disorders.

## CONCLUSION

The present investigation suggests that the otoacoustic emission contralateral inhibition failure test by a contralateral auditory stimulus be predictive of school performance disorder in individuals between six and twelve years of age.
